# Mr. Simmons, on the Plaster-Bandage

**Published:** 1808-09

**Authors:** W. Simmons

**Affiliations:** Manchester


### *To the Editors of the Medical and Phyſical Journal.*

Gentlemen,

In the Number of your Journal for February 1803, you were pleased to give insertion to my 'Observations on the Use of the Plaster-bandage in Ulcers?' In these observations I had stated my conviction of the utility of that mode of treatment, in several different kinds of ulcers; and had endeavoured to point out the distinctions which are necessary to success in its proper application. My experience since that time, has not enabled me to make any additional remark of importance; but it has confirmed those which I had formerly offered; and with me the practice is now fully established.

In noticing the different cases, in which the plaster-bandage might be applied, it was foreign to my intention to specify those of an opposite description. One of these, however, I shall now take occasion to mention. Where there is a disposition to gangrene in an ulcer; or where in a recent wound a similar tendency is manifested, pressure will prove injurious; and therefore until 'after the subsidence sidence of the inflammation,' and the parts be reduced to the state of 'a simple ulcer,' the plaster-bandage should be discontinued. In order to this effect, the free exhibition of bark, wine, and opium, will be found expedient, as in cases of erysipelas and gangrene, from an internal cause; and topically, instead of the plaster-bandage, soothing applications are indicated; such as the common cataplasm, with or without opium: or, if the discharge is fetid, the effervescing poultice, or what, in my opinion, is still better, the common poultice, with an admixture of charcoal in fine powder.

I have been induced to make these observations, because they confirm on shore, what is stated in your last Number (p. 110), by your correspondent Mr. Warnock, relative to the misapplication of the plaster-bandage on board a ship; and, by suggesting more caution, to guard against the indiscriminate employment of it.

### PAPER-BANDAGE.

To prevent disappointment in those who have not made the trial, I have also to communicate the result of my experience of the strips of adhesive plaster, when spread upon *cartridge-paper*, as a substitute for linen; and I am sorry to add, that it has not answered the expectations which I had been led to form of its utility.

In the cases of hospital practice, in in-patients, *where absolute rest was enjoined*, the paper appeared to be not inferior in efficacy to calico or linen; but, in out-patients, the experiment has failed. In this class of patients, which constitutes by far the most numerous one, the friction of the paper, from its unyielding quality, on motion of the limb, has so irritated and inflamed the part, as to cause the spreading of the ulcer, and, for a time, prohibit its use altogether. In other instances, the very opposite to this has rendered the application useless. For in dyers, and such like branches of trade, where wet or moisture is predominant, softened as the paper must be by water in the pursuit of their laborious occupation, it has ceased to operate, not only as a bandage, but even as a *defensive* covering.

A profuse limpid discharge from an ulcer, would raise a similar objection: not to insist on the frequent tearing of the paper, even in a dry state, in rude attempts to produce the requisite degree of compression over the ulcerated surface.

In

In incurable ulcers on the lower extremites, where the alleviation of the pain and mitigation of the sufferings of the patient can alone be expected, the above objections will apply with nearly equal force. And when we consider, that, on a moderate computation, an in-patient will cost a charity twelve shillings per week, while, in an out-patient, the expence will average little more than the price of the calico on which the plaster is spread; on the score of economy, a comparison can hardly be instituted. And I have not observed moderate exercise to be detrimental to the patient, or to retard the healing of the ulcer, where the limb had been supported by the cloth adhesive bandage.

I am, &c.

W. SIMMONS.

*Manchester, August* 6, 1808.

